# Short-term perceived quality of life after surgical resection for benign tracheal stenosis: a pre-post intervention study

**DOI:** 10.1093/icvts/ivaf090

**Published:** 2025-04-11

**Authors:** Vittorio Aprile, Diana Bacchin, Alessandra Lenzini, Maria Giovanna Mastromarino, Stylianos Korasidis, Sara Gabriele, Alessandro Ribechini, Mario Milazzo, Marcello Carlo Ambrogi, Marco Lucchi

**Affiliations:** Division of Thoracic Surgery, Cardiac, Thoracic, and Vascular Department, University Hospital of Pisa, Pisa, Italy; Department of Surgical, Medical and Molecular Pathology and Critical Care Medicine, University of Pisa, Pisa, Italy; Department of Surgical, Medical and Molecular Pathology and Critical Care Medicine, University of Pisa, Pisa, Italy; Division of Thoracic Surgery, Cardiac, Thoracic, and Vascular Department, University Hospital of Pisa, Pisa, Italy; Department of Surgical, Medical and Molecular Pathology and Critical Care Medicine, University of Pisa, Pisa, Italy; Division of Thoracic Surgery, Cardiac, Thoracic, and Vascular Department, University Hospital of Pisa, Pisa, Italy; Division of Thoracic Surgery, Cardiac, Thoracic, and Vascular Department, University Hospital of Pisa, Pisa, Italy; Department of Surgical, Medical and Molecular Pathology and Critical Care Medicine, University of Pisa, Pisa, Italy; Division of Thoracic Surgery, Cardiac, Thoracic, and Vascular Department, University Hospital of Pisa, Pisa, Italy; Department of Civil and Industrial Engineering, University of Pisa, Pisa, Italy; Division of Thoracic Surgery, Cardiac, Thoracic, and Vascular Department, University Hospital of Pisa, Pisa, Italy; Department of Surgical, Medical and Molecular Pathology and Critical Care Medicine, University of Pisa, Pisa, Italy; Division of Thoracic Surgery, Cardiac, Thoracic, and Vascular Department, University Hospital of Pisa, Pisa, Italy; Department of Surgical, Medical and Molecular Pathology and Critical Care Medicine, University of Pisa, Pisa, Italy

**Keywords:** tracheal surgery, tracheal anastomosis, quality of life, post-operative abilities, tracheal benign stenosis

## Abstract

**OBJECTIVES:**

Benign tracheal stenosis, as a complication of intubation or tracheotomy, is a rare but life-threatening condition. Surgical resection with end-to-end anastomosis is considered the standard treatment, when possible, providing satisfactory results in over 90% of cases. However, limited research has focused on assessing the subjective perception of quality of life (QoL) following this surgical intervention.

**METHODS:**

This study involved patients who underwent surgical treatment for tracheal stenosis at the Thoracic Surgery Unit of Pisa over a 10-year period, including 12 patients treated after March 2020 during the peak of the Covid-19 pandemic. A dedicated mixed-method questionnaire was administered to evaluate short-term perceived QoL across three domains: general functionality, organ-specific functionality and psychological well-being. The assessment was performed both before surgery (PRE-period) and 3 months after surgery (POST-period).

**RESULTS:**

The study included 22 patients and found a significant overall improvement in QoL following surgery, with a mean total score reduction of −14.64 (95% CI: −18.45 to 10.90, *P* < 0.001). General functionality and psychological well-being scores improved notably, with POST-PRE differences of −7.59 (95% CI: −9.22 to 5.68, *P* = 0.015) and −3.18 (95% CI: −4.22 to 1.91; *P* = 0.046), respectively. Patients with a history of Covid-19 showed greater improvements in general functionality (*P* = 0.042) and psychological well-being (*P* = 0.043) than others.

**CONCLUSIONS:**

Surgical intervention for tracheal stenosis significantly enhances perceived patients’ QoL, particularly in general functionality and psychological well-being areas. The results indicate that patients with a history of Covid-19 may experience a more pronounced recovery. Despite the risk of early postoperative complications, the overall improvement in QoL supports the effectiveness of surgical treatment.

## GRAPHICAL ABSTRACT

**Figure:**
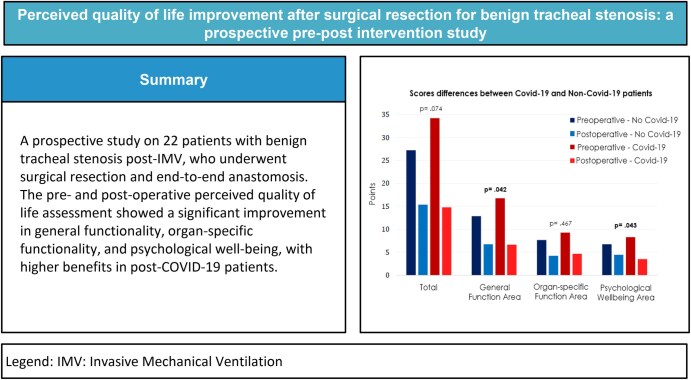

## INTRODUCTION

Benign tracheal stenosis (BTS) is a serious and potentially life-threatening condition that arises, in most cases, as a complication of invasive mechanical ventilation (IMV), such as oro-tracheal intubation and tracheotomy [1]. Mechanical ventilation is a critical intervention in patients with respiratory failure, although it can lead to significant long-term complications, including BTS that can be found in up to 20% of cases following prolonged intubation or tracheotomy [2]. This condition results in an increasing narrowing of the tracheal space, which can severely impair respiratory function and the quality of life (QoL) [3].

The treatment of tracheal stenosis depends on several factors, including the severity and the site of the stenosis, the results of previous treatments, the time of recurrence in case of conservative approaches and the clinical status of each patient [4]. Management strategies for BTS range from endoscopic interventions, such as dilatation or laser therapy, to more definitive surgical procedures [5]. Surgical treatment often involves tracheal resection with end-to-end anastomosis, where the stenotic segment of the trachea is removed, and the healthy ends are reconnected to guarantee an airway adequate caliber [6]. While surgical intervention is generally considered for severe cases or when other treatments have failed, it is associated with significant risks and requires careful consideration of the patient’s condition [7].

Tracheal stenosis has profound physical and clinical implications, including difficulty in breathing, speaking and swallowing, as much as recurrent respiratory infections requiring repeated medical interventions (especially in patients with tracheostomy). Beyond these physical health challenges, BTS can negatively affect QoL by impairing the ability of carrying out normal daily activities. Despite the recognized importance of QoL in the management of chronic conditions, there has been little research investigating how tracheal resection surgery influences QoL in patients with BTS.

Understanding this aspect is crucial, as QoL is a key outcome measure that reflects the overall well-being of patients and their ability to resume normal activities in the post-treatment phase. This study aims to fill the existing knowledge gap by assessing the perceived QoL in patients with BTS before and after tracheal resection surgery.

## MATERIALS AND METHODS

### Study design

This study was designed as a retrospective analysis of monocentric prospective cohort study conducted over a 10-year period, from January 2012 to December 2021 on all patients who underwent tracheal resection followed by end-to-end anastomosis due to benign stenosis. All patients gave full consent regarding data collection and its use in clinical studies; moreover, they gave their consent to update database information. Data were acquired from a prospective database, and all follow-up information was collected from medical reports.

### Inclusion criteria

This study focused on patients diagnosed with BTS as a complication of IMV regardless of the underlying condition that necessitated it. Only patients who underwent surgical treatment involving tracheal resection followed by end-to-end anastomosis were included. All patients enrolled in the study had completed the questionnaire both prior to surgery and 3 months postoperatively. All cases in which it was not possible to administer the questionnaire either before or after surgery were excluded due to the lack of data necessary for comparison. A written informed consent was achieved from all participants.

### Exclusion criteria

Patients with tracheal stenosis caused by malignant conditions were excluded. Malignant tracheal stenosis often involves complex treatment protocols and has different outcomes compared to benign stenosis, which could confound the assessment of QoL. Patients who were treated solely with endoscopic procedures, without undergoing tracheal resection surgery, were excluded. This criterion was set to specifically evaluate the impact of surgical resection on QoL. Lastly, patients with tracheo-oesophageal fistula were excluded from the study.

### Pre-operative work-up

**Figure 1:**
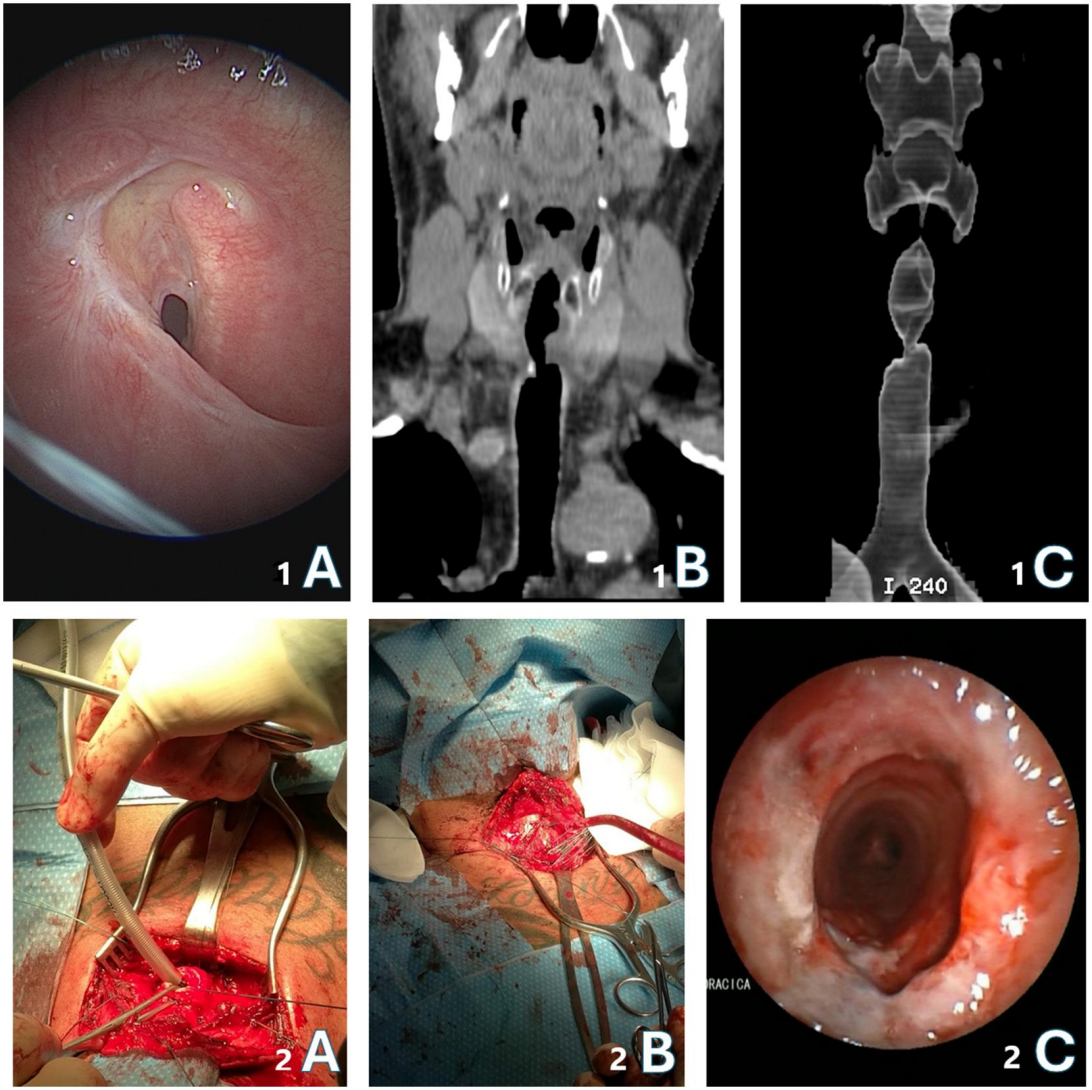
(**1A**) Endoscopic image of a severe subglottic tracheal stenosis; (**1B**) CT coronal view of the tracheal stenosis; (**1C**) 3D cervical reconstruction of upper airway area; (**2A**) intraoperative cross-field ventilation; (**2B**) intraoperative picture of tracheal anastomosis by single stiches; (**2C**) post-operative endoscopic check of trachea resection

All patients included in the study were pre-operatively evaluated in a multidisciplinary board including a thoracic endoscopist, a thoracic surgeon, a pneumologist and an anesthesiologist. Patients had a preoperative endoscopic evaluation by flexible bronchoscopy to assess the anatomical landmarks of the stenosis and the residual tracheal caliber, as well as a computed tomography (CT) scan with coronal and 3D reconstruction to better plan the surgical procedure and assess relationships with nearby tracheal structures as reported in Fig. 1. The details of the surgical procedure have been described in previous articles published by our group [6].

Specifically, the principal objective of this study was comparing preoperative and postoperative perceived QoL outcomes to evaluate the impact of the surgery by identifying any significant improvement in general function, organ-specific function, psychological well-being and overall QoL. Other clinical assessments, such as spirometry or swallowing evaluations, were performed not routinely but only when clinically indicated for the patient. Therefore, they were not included in this study. The study aimed to identify variables influencing outcomes, such as age, stenosis severity, ventilation duration and comorbidities, to determine factors most associated with changes in QoL scores. This could help pinpoint patient groups benefiting most from surgery and suggest improvements in management. Data collection included baseline demographics, stenosis characteristics, ventilation duration, comorbidities and prior treatments. Intraoperative details covered resection extent, surgery duration and complications, while postoperative data focused on recovery, complications and additional interventions.

### QoL assessment

The assessment of QoL was conducted using a purpose-built quali-quantitative questionnaire (Table 1), specifically designed. This questionnaire was based on the EuroQoL instruments, widely used for evaluating health-related QoL [8]. This questionnaire was specifically adapted for this study and was administered preoperatively and again 3 months post-surgery, this latter either through a phone call or during an outpatient clinic visit, depending on each patient’s preference and convenience. For each functional area, patients answered three or four questions, giving a score from 1 (no symptoms) to 5 (highest level perceived). Only the pain assessment was rated on a scale from 1 to 10, for continuity with the Numeric Rating pain Scale (NRS) commonly used in clinical practice. Consequently, a higher score on the questionnaire corresponded to a worse QoL, with a maximum potential score of 55/55 and a minimum score of 10/55. Finally, patients were asked to express their personal satisfaction with the intervention on a scale from 0 (not at all satisfied) to 5 (completely satisfied) and to indicate in months the time required to return to a life condition perceived as ‘normal’ (prior to stenosis).

**Table 1:** The QoL questionnaire

	Questions ranging from 1 (min.) to 5 (max)
General function area (20 points)	Did you have difficulties or feel out of breath (1–5): climbing stairs? walking certain distances on foot? washing, combing hair, dressing? carrying out normal activities or work?
Organ-specific function area (15 points)	This section assessed difficulties (1–5): speaking? breathing? swallowing food or drinks?
Psychological well-being area (20 points)	This area evaluated the psychological impact of the condition, focusing on: the experience of pain (1–10) anxiety, thoughts or worries related to the condition (1–5) fear of feeling unwell due to the condition (1–5)
Subjective evaluation	time to feel a complete return to a life they could define as ‘normal’ (in months) satisfaction from surgery? (0–5)

### Statistical analysis

The statistical analysis was performed using SPSS software (version 23). The Shapiro–Wilk normality test was applied to assess the distribution of continuous variables. Continuous variables were expressed as mean and standard deviation (SD) for normally distributed data or as median and interquartile range (IQR) for non-normally distributed data. Comparisons between continuous variables were conducted using the Student’s t-test for normally distributed data and the Mann–Whitney U-test for non-normally distributed data. Categorical variables were described as absolute numbers and percentages and compared using the chi-square test or Fisher’s exact test, as appropriate. Differences among multiple groups were evaluated using a one-way analysis of variance (ANOVA) or the Kruskal–Wallis test, depending on the normality of the data. Additionally, subgroup analyses were adjusted for multiple comparisons using the Bonferroni correction. The adjusted significance level was set according to the number of comparisons performed. Potential confounding factors, including gender, age, clinical condition, comorbidities, prior Covid-19 infection, tracheostomy and post-operative complications, were examined for their impact on QoL outcomes through a linear regression analysis, setting the investigated factor as the independent variable and the change in QoL score as the dependent variable. Characteristics of stenosis, such as diameter, length and distance from the glottis, were also included in the analysis. Statistical significance was set at *P* < 0.05, unless otherwise specified after applying the Bonferroni correction.

## RESULTS

A total of 37 patients with BTS underwent tracheal resection with end-to-end anastomosis over a 10-year period. Of these, 22 patients (59.5%) completed both pre- and post-surgical QoL questionnaires and were included in the final analysis. The remaining 15 patients (40.5%) were excluded due to refusal to participate (13 cases) or death before the 3-month follow-up (2 cases).

All patients underwent tracheal resection followed by end-to-end anastomosis as reported in a previous study by a cervical approach [6, 9]. Seven patients (31.8%) developed early complications, while one patient (4.5%) experienced a recurrence of stenosis requiring endoscopic balloon dilatations as reported in Supplementary Table S1.

### QoL evaluation

**Figure 2:**
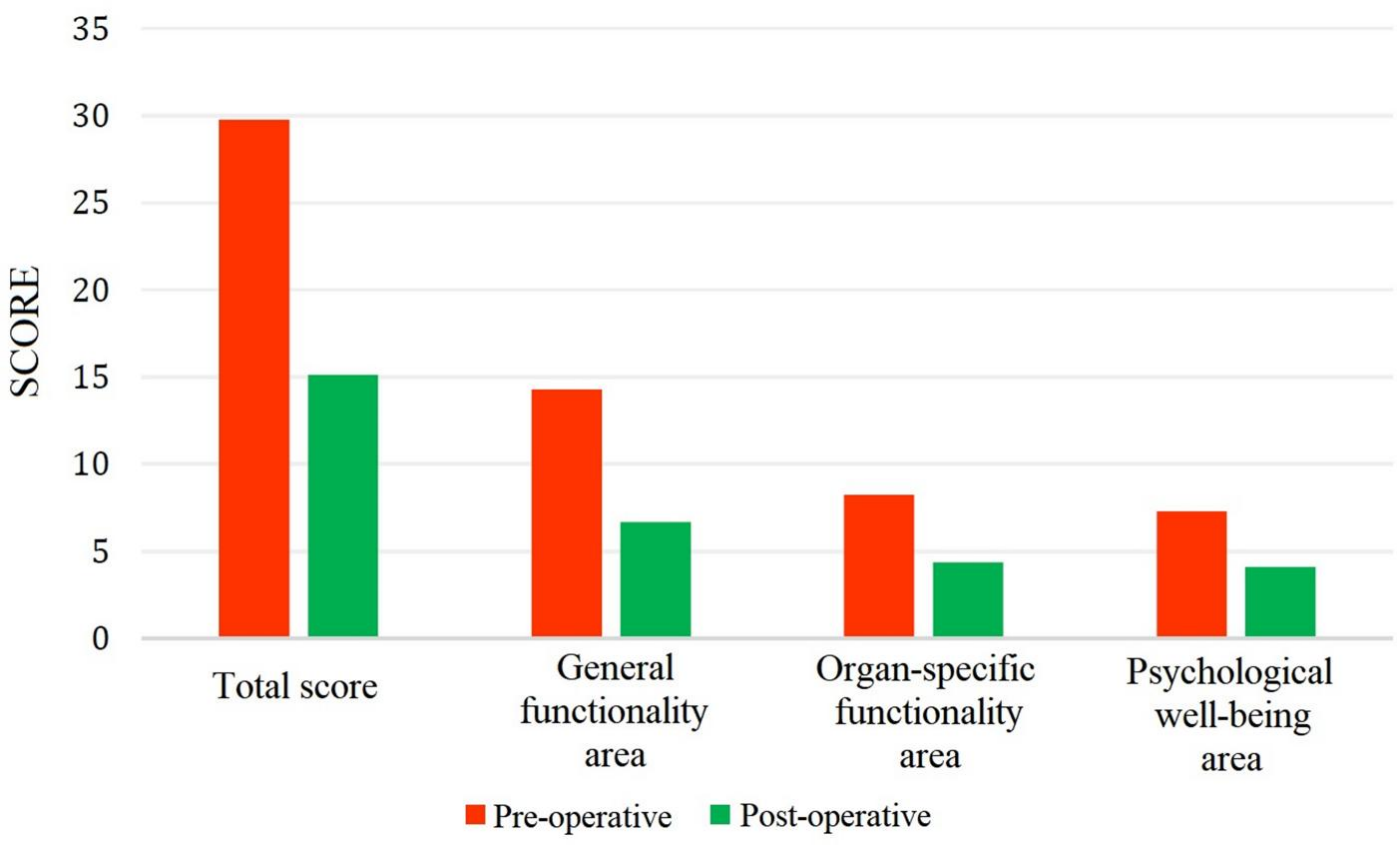
Total scores and area-specific scores obtained in the questionary with reference to the PRE-operative and POST-operative period

The analysis of the total scores revealed a significant improvement in patients’ perceived QoL following the surgical intervention as reported in Fig. 2 and in Supplementary Table S2. The median time perceived as necessary to return to a ‘normal’ life after tracheal resection surgery was 2 months (IQR: 1.0–5.75). Remarkably, all patients reported full satisfaction with their surgical experience (5/5), indicating a high level of contentment with the outcomes of the procedure and the subsequent improvement in their QoL.

The analysis of QoL scores revealed a significant improvement following surgical intervention. Preoperatively, patients reported a mean total score of 29.77 ± 9.54, indicating a considerable burden of symptoms and limitations. When broken down into specific domains, preoperative scores demonstrated impairments in general functionality (14.27 ± 4.87), organ-specific functionality (8.23 ± 3.46) and psychological well-being (7.27 ± 2.95). At 3 months post-surgery, all domains showed marked improvement. The mean total score decreased significantly to 15.14 ± 6.67 (mean difference POST-PRE: −14.64 ± 9.68, 95% CI: −18.45 to 10.90, *P* < 0.001), with general functionality improving to 6.68 ± 4.10 (mean difference POST-PRE: −7.59 ± 4.49, 95% CI: −9.22 to 5.68, *P* = 0.015), organ-specific functionality to 4.36 ± 1.84 (mean difference POST-PRE: −3.86 ± 3.60, 95% CI: −5.18 to 2.40, *P* = 0.402) and psychological well-being to 4.10 ± 2.79 (mean difference POST-PRE: −3.18 ± 2.79, 95% CI: −4.22 to 1.91, *P* = 0.046). These improvements underscore the positive impact of restoring airway patency, leading to significant enhancements in both physical functionality and psychological health.

**Figure 3:**
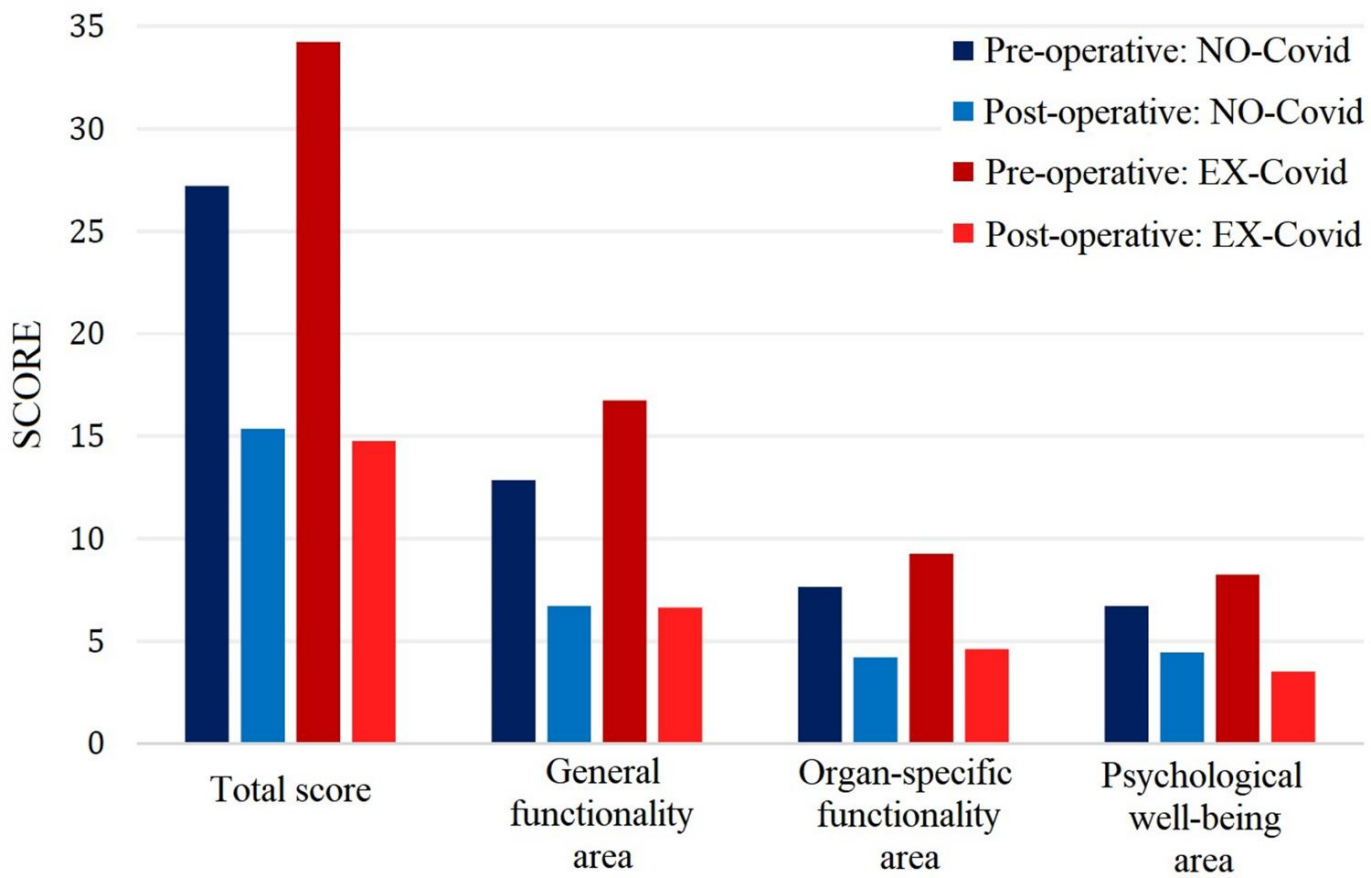
Total and area-specific scores obtained in the questionnaire referred to the PRE-operative and POST-operative period, in relation to patients with a history of SARS-CoV-2 infection (EX-Covid) and patients without a history of infection (NON-Covid)

As reported in Fig. 3 and in Supplementary Table S3, the study also analysed the impact of Covid-19 pneumonia on the recovery outcomes by comparing the results between two groups: patients who had previously contracted Covid-19 (EX-Covid group) and those who had not (NON-Covid group). Notably, the EX-Covid group, if compared to other patients, demonstrated greater relative improvements in both general functionality (mean difference POST-PRE: −10.12 ± 4.26 vs −6.14 ± 4.07, *P* = 0.042) and psychological (mean difference POST-PRE: −4.75 ± vs −2.29 ± 2.67, *P* = 0.043) areas after surgery. The relationship between previous Covid-19 and the abovementioned outcomes was confirmed by the linear regression analysis (for general functionality: β= −3.98, SE: 1.81, 95% CI: −7.66 to 0.42, *P* = 0.043; for psychological well-being: β= −2.46, SE: 1.06, 95% CI: −4.45 to 0.45, *P* = 0.036). This suggests that patients who had experienced Covid-19, possibly due to the severe respiratory compromise associated with the virus infection, may have had an increased perception of benefit from the surgical intervention. The more pronounced improvement in their QoL could also be attributed to a broader recovery from both tracheal stenosis and the lingering effects of Covid-19, reflecting a more comprehensive restoration of both physical and psychological health.

### Analysis of potential factors affecting the perceived QoL

**Figure 4:**
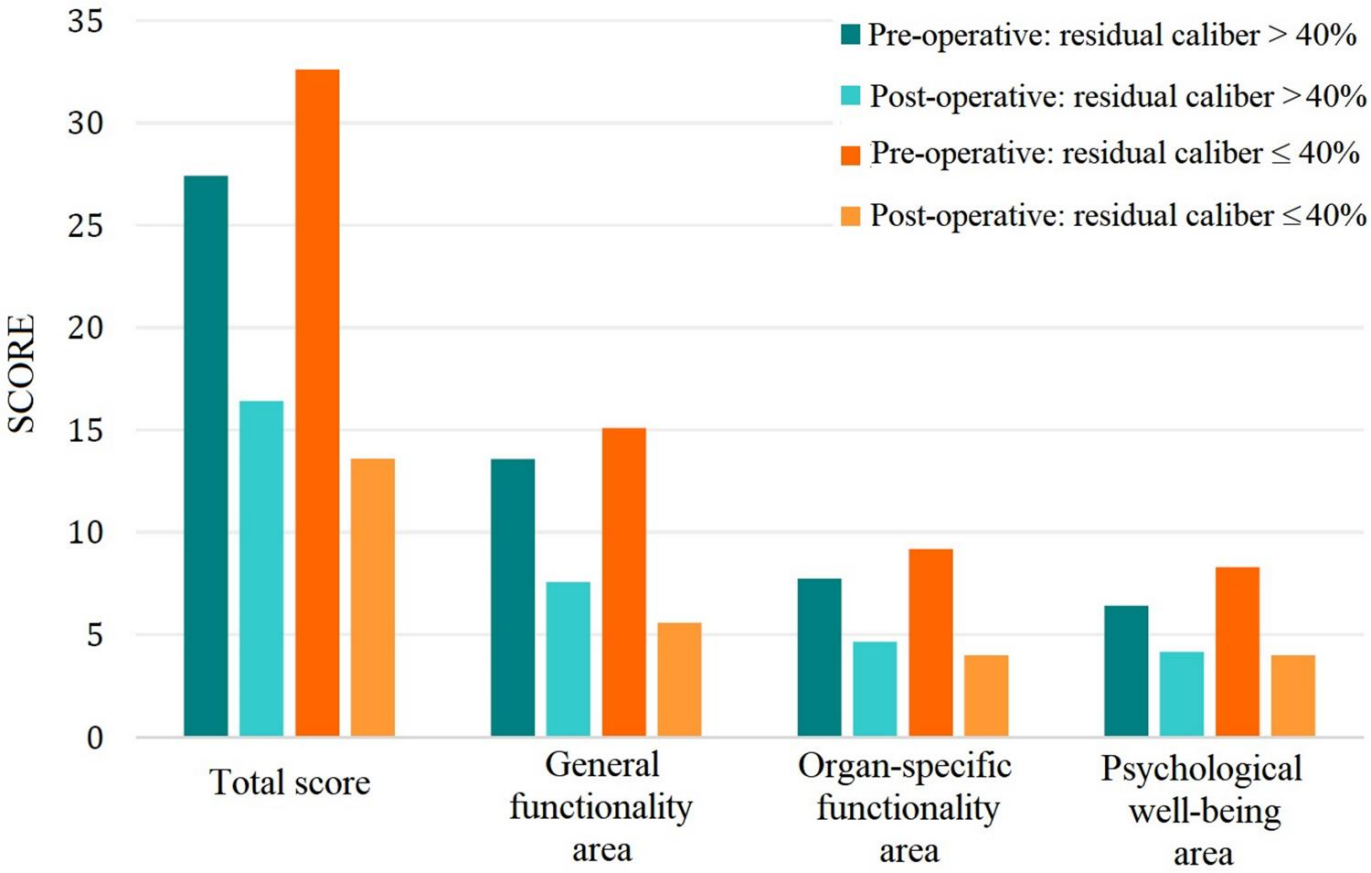
Total and area-specific scores obtained in the questionnaire referred to PRE-operative and POST-operative period, in relation to patients with a residual tracheal caliber ≤ 40% and patients with a residual caliber >40%

Most of the patients interviewed (20, 90.9%) reported an improvement in their QoL. Therefore, we aimed to investigate the factors that may have influenced this outcome. Regarding the characteristics of the stenosis, the average scores of the PRE and POST periods were compared, as were the differences in means between patients with a residual caliber ≤40% (the median of our cohort) and those with a residual caliber >40%, a critical threshold where patients begin to experience dyspnoea even at rest as reported in Fig. 4. Specifically, the difference between total scores was found to be significant (mean difference PRE-POST: −19.00 ± 3.02 in case of residual lumen ≤40% vs −11.00 ± 2.21 for residual lumen >40%, 95% CI: −0.65 to 15.31, *P* = 0.041), with β= −8.00, SE: 3.85, 95% CI: −15.63 to 0.27, *P* = 0.049. However, the differences in individual areas were not significant. Moreover, patients who have had a tracheotomy also reported an improvement trend in every explored area (mean difference PRE-POST: −12.73 ± 2.63 vs 18.71 ± 2.37, 95% CI: −13.02 to 1.22, *P* = 0.061 for global score, −6.86 ± 1.23 vs −9.14 ± 1.01, 95% CI: −5.33 to 1.13, *P* = 0.073 for the general function area score, −3.13 ± 0.92 vs −5.43 ± 1.04, 95% CI: −5.16 to 1.15, *P* = 0.027 for the organ-specific function area score, −2.73 ± 0.78 vs −4.14 ± 0.67, 95% CI: −3.58 to 0.12, *P* = 0.052 for the psychological well-being area score).

Conversely, no significant differences were observed in any of the three areas for the following analysed factors: gender, age, ECOG score, presence of concomitant diseases, other tracheal stenosis anatomical features, previous and the number of endoscopic treatments, time between the creation of the tracheotomy or IMV and the surgical procedure, or the occurrence of postoperative complications.

## DISCUSSION

Undoubtedly, dyspnoea (experienced as the inability to breathe adequately, to modulate breathing according to one’s needs, and the sensation of shortness of breath or suffocation) represents one of the most distressing and potentially life-threatening events a person may face [10]. To date, numerous studies have been conducted on the QoL in patients with head and neck cancers or those who have undergone laryngectomy [11, 12]. However, functional outcomes following tracheal surgery for benign laryngotracheal stenosis are described in only a limited number of studies [13–15].

Clunie *et al.* conducted a systematic review of studies that reported voice and swallowing outcomes in adults undergoing reconstructive surgery for laryngotracheal stenosis. They concluded that swallowing function is more likely to return to pre-surgical levels than voice function [13]. Similarly, Timman *et al.* [14] investigated functional outcomes after (laryngo)tracheal resection and reconstruction for acquired benign (laryngo)tracheal stenosis, with particular focus on the impact of laryngeal involvement. They employed various questionnaires to assess the impact of surgery on QoL, such as the EuroQol five-dimensions questionnaire (EQ-5D), which evaluates mobility, self-care, usual activities, pain or discomfort and anxiety or depression. Additionally, they used the EuroQol visual analogue scale (VAS) to document patients’ self-reported health status and the Voice Handicap Index (VHI) to assess phonation impairment. However, their study included patients who underwent different surgical procedures with various approaches and outcomes, and the questionnaires were administered only after a mean follow-up period of over 5 years post-surgery.

More recently, Nauta *et al.* [4] reported their experience with laryngotracheal resection and anastomosis for benign stenosis, focusing on postoperative QoL as assessed by functional questionnaires, including the VAS and the modified Medical Research Council scale for dyspnoea, a VAS for swallowing, and the VHI. The authors documented a significant improvement in dyspnoea as assessed by the VAS dyspnoea scores; however, VAS dysphagia scores did not differ significantly after surgery compared to baseline. Additionally, only 12% of patients had VHI values indicative of severe voice impairment.

This study provides valuable insights into the QoL outcomes for patients undergoing tracheal resection with end-to-end anastomosis for BTS, assessed both before and after surgery. The findings highlight the significant impact of this condition on both physical functioning and psychological well-being, underscoring the comprehensive burden experienced by affected individuals.

Overall, the study demonstrates that surgical intervention for tracheal stenosis is highly effective in improving patients’ QoL. The substantial reduction in total and specific domain scores illustrates the broad benefits of treatment, particularly in enhancing general daily functioning and psychological well-being. These findings emphasize the importance of a multidisciplinary approach that not only focuses on the physical resolution of tracheal obstruction but also addresses the emotional and psychological aspects of recovery [15]. The notable improvements in psychological well-being suggest that alleviating the fear and anxiety associated with respiratory distress and the potential for airway obstruction can lead to better overall patient outcomes [16, 17].

### Impact of tracheal stenosis on QoL

The study shows that tracheal stenosis significantly affects patients’ QoL, both physically and psychologically. Patients face considerable challenges in daily activities like climbing stairs, walking and self-care due to respiratory issues and frequent infections. Additionally, the psychological impact is severe, with patients experiencing high levels of anxiety, fear and emotional distress, driven by the life-threatening nature of BTS and the need for repeated medical interventions. These findings align with previous research highlighting the profound physical and emotional toll of chronic respiratory diseases [18, 19].

Our findings also indicate that patients with more severe stenosis (residual lumen <40%) and those who had undergone tracheotomy before surgery experienced greater improvements in QoL postoperatively. This may be attributed to the substantial relief of symptoms that these patients experience after surgery, as the resection significantly alleviates the critical narrowing of the airway. Patients who undergo tracheostomy often face additional challenges, including difficulties with speech and an increased risk of infections, which might explain the greater perceived benefit following successful surgical intervention.

### Impact of Covid-19 on patients with tracheal stenosis

Interestingly, our study found that patients who developed tracheal stenosis following IMV due to Covid-19 pneumonia reported more severe impairment in QoL. This finding may be related to the broader psychological impact of the Covid19 pandemic. The pandemic has been associated with increased levels of anxiety, depression and post-traumatic stress, particularly among individuals who have experienced severe illness or prolonged hospitalization. The stress of the pandemic, combined with the physical debilitation caused by BTS, likely compounded the psychological burden on these patients, leading to worse perceived QoL outcomes [19].

Tracheal resection followed by end-to-end anastomosis is a technically demanding procedure that carries a risk of significant morbidity. Despite these challenges, our study demonstrates that when performed by experienced surgical teams, this procedure results in substantial improvements in QoL for patients with benign BTS. The low postoperative mortality rate in our cohort and the high rate of patient satisfaction underscores the potential benefits of this surgery when carried out in specialized centers [20]. These findings are consistent with other reports in the literature, where complication rates range from 20% to 40%, highlighting the importance of surgical expertise in achieving optimal outcomes in tracheal surgery [21–23].

This study has several limitations that must be acknowledged. First, the sample size was relatively small, with only 22 patients included in the final analysis. Unfortunately, tracheal surgery is inherently associated with limited case series in the literature due to the rarity of the condition, making large-scale studies challenging. Additionally, we experienced a significant dropout rate, as it was not possible to obtain complete pre- and post-operative data for all initially enrolled patients. To evaluate the severity of tracheal stenosis as a contributing factor, we used a 40% cut-off value, which corresponds to the median stenosis severity observed in our cohort. However, this threshold was not derived from an internationally validated grading system, which may limit the generalizability of our findings. Finally, the QoL assessment questionnaire used in this study was specifically designed for our patient cohort and has not undergone formal validation, which may introduce biases and limit the external validity of our results. Despite these limitations, we believe the findings provide valuable insights into the impact of surgical intervention on QoL in patients with BTS.

## CONCLUSION

This study shows that tracheal stenosis severely affects both the physical and psychological well-being of patients. Surgical treatment, especially tracheal resection with end-to-end anastomosis, significantly improves QoL, particularly for those with severe cases or post-tracheotomy. The Covid-19 pandemic likely worsened the psychological impact of tracheal stenosis, emphasizing the need for holistic care. The study strongly supports the effectiveness of surgery in enhancing patients’ overall health and function, particularly for Covid-19 recovery patients. Due to the surgery’s complexity and risks, experienced surgical teams are essential for achieving the best outcomes.

## Supplementary Material

ivaf090_Supplementary_Data

## Data Availability

The datasets generated during and/or analysed during the current study are available from the corresponding author on reasonable request.

## Notes

Presented at the 38th EACTS Annual Meeting, Lisbon.

## ABBREVIATIONS

BTS: Benign tracheal stenosis
ECOG-PS: Eastern Cooperative Oncology Group Performance Status
IMV: Invasive mechanical ventilation
QoL: Quality of life
VCP: Vocal cord paralysis

## References

[1] Marulli G , Rizzardi G, Bortolotti L et al Single-staged laryngotracheal resection and reconstruction for benign strictures in adults. Interact CardioVasc Thorac Surg 2008;7:227–30; discussion 230. 18216046 10.1510/icvts.2007.168054

[2] Álvarez-Maldonado P , Hernández-Ríos G, Hernández-Solís A et al Tracheal resection and anastomosis in post-intubation tracheal stenosis: a systematic review. Eur J Cardiothorac Surg 2024;66:ezae330. 39254596 10.1093/ejcts/ezae330

[3] Bibas BJ , Cardoso PFG, Minamoto H et al Quality-of-life evaluation in patients with laryngotracheal diseases. Transl Cancer Res 2020;9:2099–101. 35117564 10.21037/tcr.2020.02.60 PMC8797603

[4] Nauta A , Mitilian D, Hanna A et al Long-term results and functional outcomes after surgical repair of benign laryngotracheal stenosis. Ann Thorac Surg 2021;111:1834–41. 33035455 10.1016/j.athoracsur.2020.07.046

[5] Siciliani A , Rendina EA, Ibrahim M. State of the art in tracheal surgery: a brief literature review. Multidiscip Respir Med 2018;13:34. 30214724 10.1186/s40248-018-0147-2 PMC6134582

[6] Lucchi M , Ambrogi M, Aprile V et al Laryngotracheal resection for a post-tracheotomy stenosis in a patient with coronavirus disease 2019 (COVID-19). JTCVS Tech 2020;4:360–4. 32838338 10.1016/j.xjtc.2020.08.023 PMC7423512

[7] D'Andrilli A , Maurizi G, Andreetti C et al Long-term results of laryngotracheal resection for benign stenosis from a series of 109 consecutive patients. Eur J Cardiothorac Surg 2016;50:105–9. 26792926 10.1093/ejcts/ezv471

[8] EuroQol Group. EuroQol—a new facility for the measurement of health-related quality of life. Health Policy 1990;16:199–208. 10109801 10.1016/0168-8510(90)90421-9

[9] Bacchin D , Aprile V, Lenzini A et al Surgical treatment of tracheal stenosis during Covid-19 era: a single-center experience and lessons learnt on the field. Updates Surg 2023;75:1681–90. 37458903 10.1007/s13304-023-01577-6 PMC10435409

[10] Bierbrier J , Gerstein E, Whitmore GA et al Impact of dyspnea on adults with respiratory symptoms without a defined diagnosis. Chest 2024;S0012-3692:05133–X. 10.1016/j.chest.2024.07.183 PMC11638540 39242078

[11] Chen AY , Frankowski R, Bishop-Leone J et al The development and validation of a dysphagia-specific quality-of-life questionnaire for patients with head and neck cancer: the M. D. Anderson dysphagia inventory. Arch Otolaryngol Head Neck Surg 2001;127:870–6. 11448365

[12] Lennon CJ , Gelbard A, Bartow C et al Dysphagia following airway reconstruction in adults. JAMA Otolaryngol Head Neck Surg 2016;142:20–4. 26561927 10.1001/jamaoto.2015.2562

[13] Clunie GM , Kinshuck AJ, Sandhu GS et al Voice and swallowing outcomes for adults undergoing reconstructive surgery for laryngotracheal stenosis. Curr Opin Otolaryngol Head Neck Surg 2017;25:195–9. 28277335 10.1097/MOO.0000000000000362

[14] Timman ST , Schoemaker C, Li WWL et al Functional outcome after (laryngo)tracheal resection and reconstruction for acquired benign (laryngo)tracheal stenosis. Ann Cardiothorac Surg 2018;7:227–36. 29707500 10.21037/acs.2018.03.07 PMC5900076

[15] Mahmood K , Frazer-Green L, Gonzalez AV et al Management of central airway obstruction: an American College of Chest Physicians clinical practice guideline. Chest 2025;167:283–95. 39029785 10.1016/j.chest.2024.06.3804

[16] Yohannes AM , Junkes-Cunha M, Smith J et al Management of dyspnea and anxiety in chronic obstructive pulmonary disease: a critical review. J AmMed Dir Assoc 2017;18:1096.e1–17. 10.1016/j.jamda.2017.09.007 29108885

[17] Kossyvaki V , Anagnostopoulos N, Kaltsakas G et al The value of dyspnea and spirometry in detecting relapse of benign tracheal stenosis. Respiration 2022;101:174–83. 34614495 10.1159/000519216

[18] Samad I , Akst L, Karatayli-Özgürsoy S et al Evaluation of dyspnea outcomes after endoscopic airway surgery for laryngotracheal stenosis. JAMA Otolaryngol Head Neck Surg 2016;142:1075–81. 27533026 10.1001/jamaoto.2016.2029 PMC5516931

[19] Vindegaard N , Benros ME. Covid-19 pandemic and mental health consequences: systematic review of the current evidence. Brain Behav Immun 2020;89:531–42. 32485289 10.1016/j.bbi.2020.05.048 PMC7260522

[20] Fiacchini G , Tricò D, Ribechini A et al Evaluation of the incidence and potential mechanisms of tracheal complications in patients with Covid-19. JAMA Otolaryngol Head Neck Surg 2021;147:70–6. 33211087 10.1001/jamaoto.2020.4148 PMC7677875

[21] Mammana M , Verzeletti V, Baldi M et al Surgery for tracheal and laryngotracheal stenosis: a historical case series. Eur J Cardiothorac Surg 2024;65:ezae026. 38290793 10.1093/ejcts/ezae026

[22] Abbasidezfouli A , Akbarian E, Shadmehr MB et al The etiological factors of recurrence after tracheal resection and reconstruction in post-intubation stenosis. Interact CardioVasc Thorac Surg 2009;9:446–9. 19531537 10.1510/icvts.2009.202978

[23] Krajc T , Janik M, Benej R et al Urgent segmental resection as the primary strategy in management of benign tracheal stenosis. A single center experience in 164 consecutive cases. Interact CardioVasc Thorac Surg 2009;9:983–9. 19755399 10.1510/icvts.2009.213215

