# Supplementary material for: Short-term perceived quality of life after surgical resection for benign tracheal stenosis: a pre-post intervention study
Source: Interdiscip Cardiovasc Thorac Surg. 2025 Apr 11;40(4):ivaf090. doi: 10.1093/icvts/ivaf090 (PMC12022214; doi:10.1093/icvts/ivaf090)
Supplement: ivaf090_Supplementary_Data [file ivaf090_supplementary_data.zip › Sup Table 3.docx]

|  | NON-Covid (n=14) | Ex-COVID (n=8) | *p-value* |
| --- | --- | --- | --- |
| TOTAL DIFFERENCE (MEAN, SD) | -11.86 ± 9.13 | -19.50 ± 9.15 | 0.074 |
| DIFFERENCE IN GENERAL FUNCTIONALITY AREA (meAN, SD) | -6.14 ± 4.07 | -10.12 ± 4.26 | **0.042** |
| DIFFERENCE IN ORGAN-SPECIFIC FUNCTIONALITY AREA (meAN, SD) | -3.43 ± 3.03 | -4.62 ± 4.56 | 0.467 |
| DIFFERENCE IN PSYCHOLOGICAL WELL-BEING AREA (meAN, SD) | -2.29 ± 2.67 | -4.75 ± 2.37 | **0.043** |

Supplementary Table 3. Comparison between patients who had a history of SARS-CoV-2 infection (EX-Covid) and those who had not contracted the infection (NON-Covid).

P-value: observed significance level using the one-way ANOVA test. Statistically significant differences are highlighted in bold.
