# Supplementary material for: Short-term perceived quality of life after surgical resection for benign tracheal stenosis: a pre-post intervention study
Source: Interdiscip Cardiovasc Thorac Surg. 2025 Apr 11;40(4):ivaf090. doi: 10.1093/icvts/ivaf090 (PMC12022214; doi:10.1093/icvts/ivaf090)
Supplement: ivaf090_Supplementary_Data [file ivaf090_supplementary_data.zip › Sup Table 1.docx]

| **Gender Distribution (n,%)** | - Males: 13 (59.1) - Females: 9 (40.9) |
| --- | --- |
| **Mean Age (years±SD)** | 51.4 ± 13.6 |
| **Pre-operative ECOG-PS Score (n, %)** | - 1-2: 17 (77.3) - 3-4: 5 (22.7) |
| **Median Time Between IMV and Surgery (months, IQR)** | 6 (7) |
| **Previous Tracheostomy (n, %)** | 15 (68.2) |
| **COVID-19 Pneumonia Related BTS (n, %)** | - EX- Covid group: 8 (36.4) - NON-Covid group: 14 (63.6) |
| **Previous Endoscopic Treatment* (n, %)** | 14 (63.6) |
| **Mean Residual Lumen (%±SD)** | 43.6 ± 18.2 |
| **Median Length of Stenosis (mm, IQR)** | 15 (10) |
| **Mean Distance from the Rima Glottidis (mm±SD)** | 34.5 ± 19.6 |
| **Mean Operative time (min±SD)** | 147 ± 38.4 |
| **Mean Length of Tracheal Resection (mm±SD)** | 21 ± 7.1 |
| **Laryngo-tracheal anastomosis (n, %)** | 8 (36.4) |
| **Median Hospital Stay (days, IQR)** | 9 (5) |
| **Postoperative Complications (n, %)** | 8 (36.4%)   - 4 (50%) single VCP - 1 (12.5%) Bleeding that did not require a second surgery - 1 (12.5%) Wound infection that required a second surgery - 1 (12.5%) BTS recurrence after 45 days, endoscopically treated - 1 (12.5%) partial anastomosis dehiscence conservatively treated |
| **30-day Mortality Rate (n, %)** | 0 (0%) |
| **Extubation in the Operating Room (n, %)** | 22 (100%) |
| **Post-operative ICU Stay (n, %)** | 0 (0%) |

**Supplementary Table 1:** Patient’s main clinical characteristics, tracheal stenosis features and surgical outcomes.

SD: Standard Deviation; ECOG-PS: Eastern Cooperative Oncology Group Performance Status; IMV: Invasive Mechanical ventilation; IQR: Interquartile Range; VCP: Vocal Cord Paralysis; ICU: Intensive Care Unit. *Including laser ablation, balloon dilatation and temporary stent placement.
