# Supplementary material for: Short-term perceived quality of life after surgical resection for benign tracheal stenosis: a pre-post intervention study
Source: Interdiscip Cardiovasc Thorac Surg. 2025 Apr 11;40(4):ivaf090. doi: 10.1093/icvts/ivaf090 (PMC12022214; doi:10.1093/icvts/ivaf090)
Supplement: ivaf090_Supplementary_Data [file ivaf090_supplementary_data.zip › Sup Table 2.docx]

| Area of interest |  | Scores |  | *p-value* |
| --- | --- | --- | --- | --- |
|  | Pre-operative period | Post-operative period | Mean of differences |  |
| Total scores (range, SD) | 29.77 (10–45. ± 9.54) | 15.14 (10–36. ± 6.67) | -14.64 ± 9.68 | <0.001 |
| General functionary area scores (range, SD) | 14.27 (4–20. ± 4.87) | 6.68 (4–20. ± 4.10) | -7.59 ± 4.49 | **0.015** |
| Organ-specific functionality area scores (range, SD) | 8.23 (3–15. ± 3.46) | 4.36 (3-10. ± 1.84) | -3.86 ± 3.60 | 0.402 |
| psychological weLL-being scores (range, SD) | 7.27 (3–13. ± 2.95) | 4.10 (3–13. ± 2.79) | -3.18 ± 2.79 | **0.046** |

Supplementary Table 2. Quality of Life (QoL) Outcomes

Means of the scores in relation to the pre-operative and post-operative periods, with overall total and total per area. Differences of the means between the post-operative and pre-operative periods. SD: standard deviation. P-value: observed significance level using the paired-sample Student’s t-test. Statistically significant differences are highlighted in bold.
